# Overestimated Oncologic Significance of Lymph Node Metastasis in G1 Nonfunctioning Neuroendocrine Tumor in the Left Side of the Pancreas

**DOI:** 10.1097/MD.0000000000001404

**Published:** 2015-09-11

**Authors:** Young Jin Yoo, Seok Jeong Yang, Ho Kyoung Hwang, Chang Moo Kang, Hogeun Kim, Woo Jung Lee

**Affiliations:** From the Department of Hepatobiliary and Pancreatic Surgery, Yonsei University College of Medicine (YJY, SJY, HKH, CMK, WJL); Department of Pathology, Yonsei University College of Medicine (HK); and Pancreaticobiliary Cancer Clinic, Yonsei Cancer Center, Severance Hospital, Seoul, Korea (SJY, HKH, CMK, HK, WJL).

## Abstract

Recent studies have expounded on the oncologic significance of lymph node metastasis in nonfunctioning (NF) neuroendocrine tumors (NETs) of the pancreas and suggest regional lymph node dissection for treating pancreatic NET. We tested this recommendation in NF pancreatic NET-G1, as these tumors are generally small and suitable for function-preserving minimally invasive pancreatectomy.

From January 2005 to December 2014, medical records of patients who underwent pancreatectomy for pathologically confirmed NF NET-G1 of the left side of the pancreas were retrospectively reviewed. Oncologic outcomes were compared between limited pancreatectomy and distal pancreatosplenectomy.

Thirty-five patients (14 males and 21 females) with a mean age of 55.9 ± 11.4 years were enrolled in this study. Six patients (17.1%) underwent distal pancreatosplenectomy. Limited pancreatectomies comprised 15 spleen-preserving distal pancreatectomies (42.8%), 10 enucleations (28.6%), and 4 central pancreatectomies (11.4%). Lymph node metastasis was not found in 6 patients who underwent distal pancreatectomy with a splenectomy; meanwhile, the others were regarded as pNx since no lymph node retrieval was attempted during the limited pancreatectomy. Overall disease-free survival was 36.5 months (95% confidence interval [CI]: 25.9–47.1) and no tumor-related mortality was noted. Minimally invasive pancreatectomy (*P* = 0.557) and limited pancreatectomy (*P* = 0.758) showed no adverse impact in treating NF NET-G1 of the left side of the pancreas.

The oncologic significance of lymph node metastasis is overestimated in NF NET-G1 of the left side of the pancreas. Routine conventional distal pancreatosplenectomy to retrieve regional lymph nodes may be too excessive in treating NF NET-G1 of the distal pancreas.

## INTRODUCTION

**FIGURE 1:**
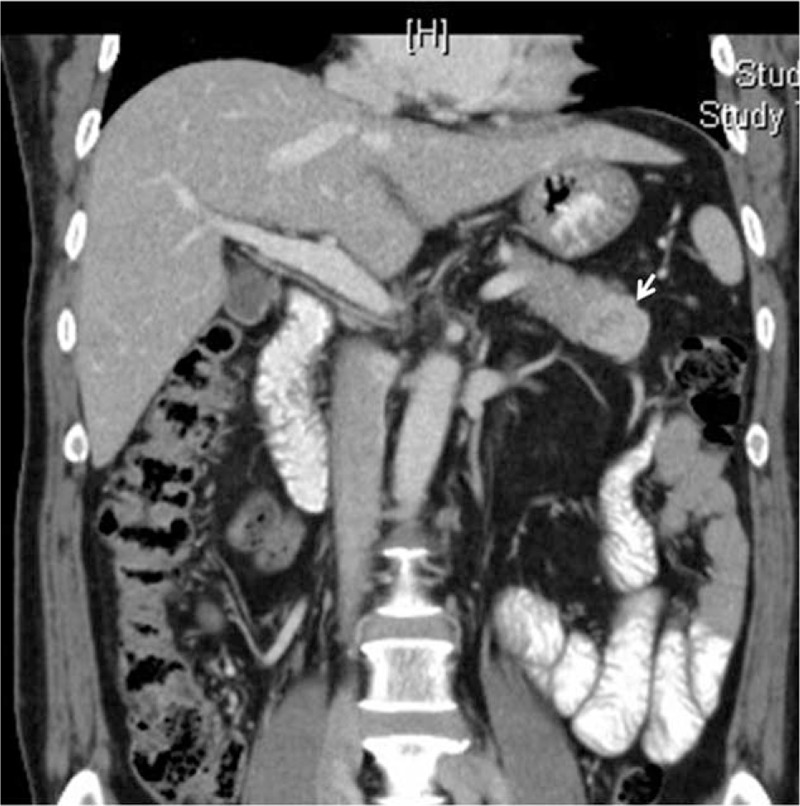
A 61-year-old male patient with NET of the left side of the pancreas (white arrow). He underwent laparoscopic spleen-preserving distal pancreatectomy. He was found to have no evidence of tumor recurrence after more than 70 months of postoperative follow-up. Pathologic diagnosis revealed a 3-cm NET-G1 with a Ki 67 index <1% and no mitosis. NET = neuroendocrine tumor.

There is some debate as to which surgical option is appropriate for treating a single nonfunctioning (NF) neuroendocrine tumor (NET) on the left side of the pancreas (as seen in Figure 1). Pancreatic NETs, known as islet cell tumors, are low grade malignant tumors that rarely occur, about 3% of all primary pancreatic neoplasms.^1^ NETs are characterized as G1 or G2 NETs and neuroendocrine carcinomas (NEC, highly malignant).^2^ In pancreatic NET, surgery is the primary treatment for localized tumors, and remains the treatment of choice for metastatic disease.^3^ Thus far, surgery has proven to be the only curative treatment thereof, providing 5-year survival rates of 80% to 100% in resectable cases.^4^

Commonly, reports suggest that NF NET of the pancreas can be removed by minimally invasive approaches (robotic or laparoscopic) and by function-preserving limited pancreatectomy, such as enucleation, central pancreatectomy, and spleen-preserving pancreatectomy.^5,6^ However, recent studies have shown that lymph node metastasis from NET varies in grade (G1, 15% to 20%; G2, 30% to 40%; G3, more than 50%)^7^ and that metastasis to lymph nodes is a poor prognostic factor.^8–10^ Therefore, regional lymph node dissection is strongly recommended in treating NET of the pancreas. Accordingly, this would mean that distal pancreatosplenectomy for lymph node retrieval would be appropriate in treating NF NET should it be found in the left side of the pancreas.

The reasons why we chose to investigate NF NET-G1 of the distal pancreas are as follows: First, greater use of more advanced imaging modalities has led to an increased incidence of asymptomatic NETs.^11,12^ Second, most NF NETs are relatively small in size.^7,13^ Third, NF NET-G1 shows quite good survival outcomes in long-term survival.^7,14^ Considering that minimally invasive enucleation, spleen-preserving distal pancreatectomy, and central pancreatectomy are regarded as safe and effective treatment options to treat benign and low-grade malignant tumors, function-preserving and minimally invasive pancreatectomy could be ideal approaches for NF NET-G1 of the distal pancreas. Lastly, laparoscopic distal pancreatectomy is generally accepted as safe and effective. On the contrary, applications of laparoscopic pancreaticoduodenectomy are still debated, and its counterpart of limited pancreatectomy (ie, duodenum-preserving pancreatic head resection) is technically demanding.

Therefore, in this study, we focused specifically on NF NET-G1 of the left side of the pancreas, for which minimally invasive and limited pancreatectomy are suitable. We evaluated oncologic outcomes of NF NET-G1s of the left side of the pancreas according to clinicopathological factors, surgical approach, and extent of pancreatectomy to suggest the role of minimally invasive and function-preserving limited pancreatectomy in treatment thereof.

## METHODS

**FIGURE 2:**
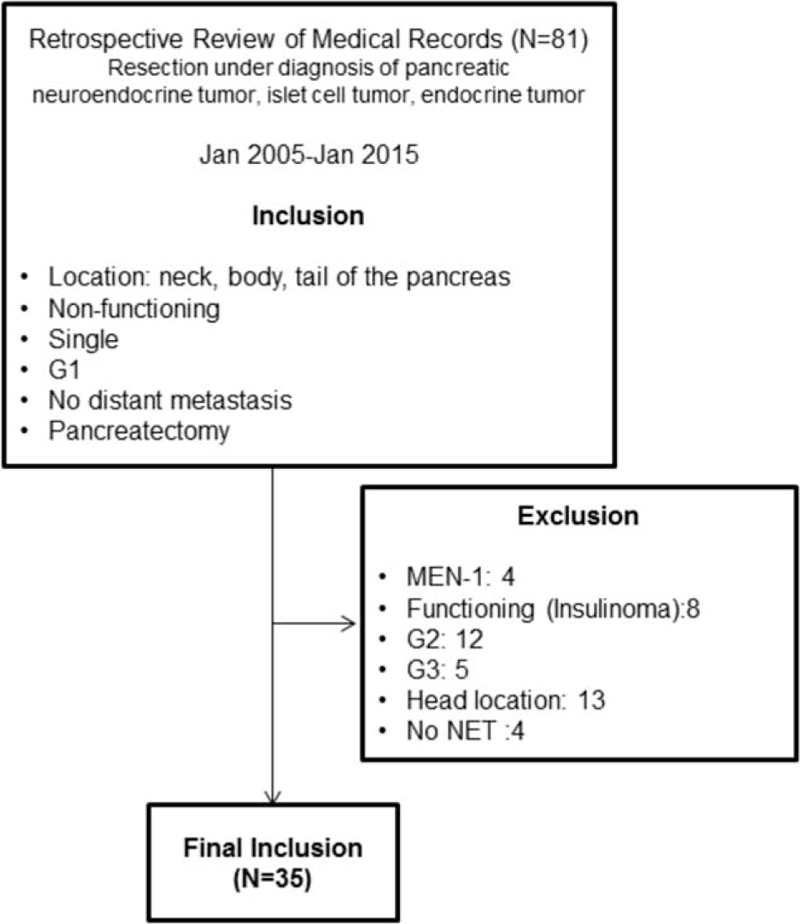
Diagram for study enrollment.

This study was approved by our institutional review board, and informed consent was not required. From January 2005 to December 2014, the medical records of patients who underwent pancreatectomy for a pathological diagnosis of NET of the left side of the pancreas were retrospectively reviewed. Only patients fulfilling inclusion criteria were included in this study (criteria are shown in Table 1 and Figure 2). Clinicopathological variables, such as age, gender, symptom, radiologic tumor size, surgical procedures, grade, Ki-67 proliferative index, complications, recurrence, and disease-specific mortality, were checked. A specialized pathologist re-evaluated tumor grades by performing Ki-67 staining and mitotic counts according to the World Health Organization (WHO) grading system for NET.^9^ pN-stage was determined based on complete pathological examination of a conventional distal pancreatosplenectomy. In cases of limited pancreatectomy, pN stage was regarded as pNx, because regional lymph nodes were not retrieved.

**TABLE 1:**
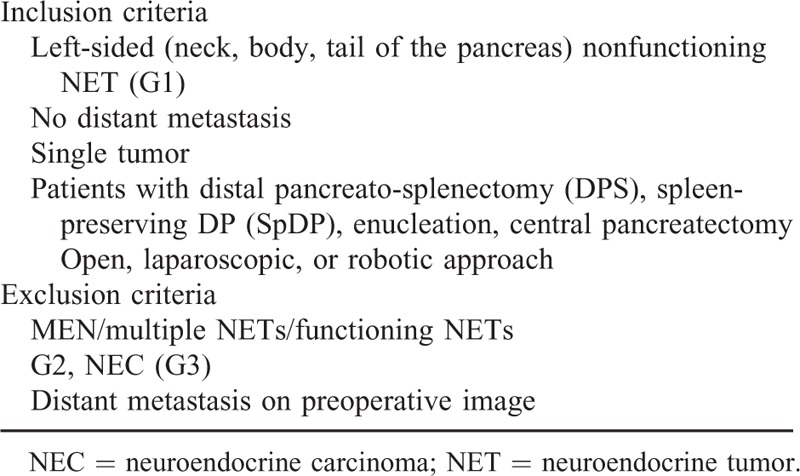
Inclusion and Exclusion Criteria

### Statistical Analyses

Continuous variables are described as mean ± standard deviation, and categorical variables are described as frequencies (%). Student *t* test and chi-squared test or Fisher's exact test were applied to compare continuous and categorical variables, respectively. Survival curves were estimated using the Kaplan–Meier method to calculate cumulative disease-free survival and disease-specific survival rates. Statistical analyses were performed using SPSS 20.0 for Windows (SPSS Inc., Chicago, IL). All *P* values < 0.05 were considered statistically significant.

## RESULTS

### Patient Characteristics

**FIGURE 3:**
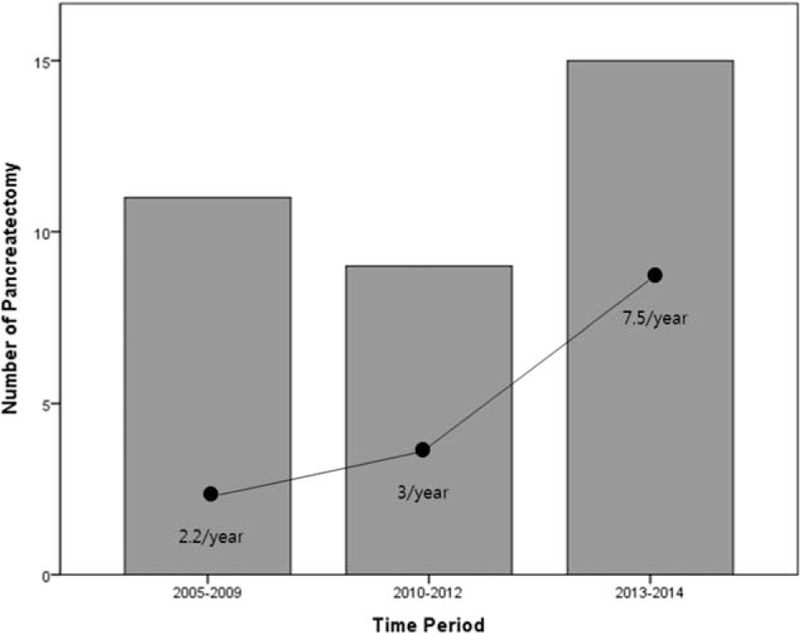
Incidence of pancreatectomy for NF NET-G1 of the left side of the pancreas. NET = neuroendocrine tumor; NF = nonfunctioning.

Throughout the study period, the incidence of pancreatectomy for NF NET-G1 of the pancreas increased (Figure 3). Thirty-five patients (14 males and 21 females), with a mean age of 55.9 ± 11.4 years, were identified with a pathologically confirmed diagnosis of grade 1 NF neuroendocrine (islet cell) tumor (NF NET-G1) of the left side of the pancreas. An incidental diagnosis was noted in most patients (29 out of 35 patients, 82.9%), and abdominal discomfort was the most common symptom. Five patients (14.3%) had NET in the neck portion of the pancreas, 17 (48.6%) in the body, and 13 (37.1%) in the tail, with a radiologic tumor size of 1.8 ± 0.9 cm. Tumors showed well demarcated margins, and there was no radiologic evidence of local tumor invasion.

Fifteen patients (42.8%) underwent spleen-preserving distal pancreatectomy, while distal pancreatosplenectomy was performed in 6 patients (17.1%), enucleation in 10 patients (28.6%), and central pancreatectomy in 4 patients (11.4%, Table 2). Minimally invasive pancreatectomy was more frequently applied to treat NF NET-G1 of the distal pancreas (*P* = 0.025); however, there was no difference in rate of limited pancreatectomy (*P* = 1.000, Table 3). Lymph node metastasis was not found in 6 patients with distal pancreatectomy with splenectomy; all others were regarded as Nx since lymph node retrieval was not attempted during limited pancreatectomy.

**TABLE 2:**
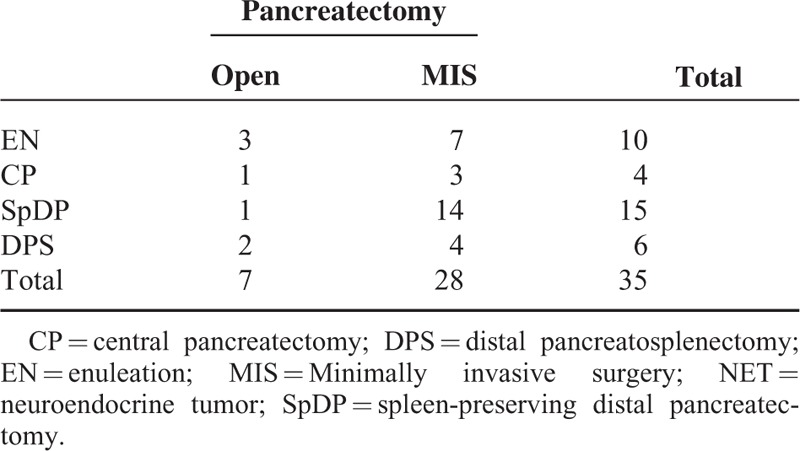
Characteristics of Pancreatectomy to Treat G1 NF-NET of the Left-Sided Pancreas

**TABLE 3:**
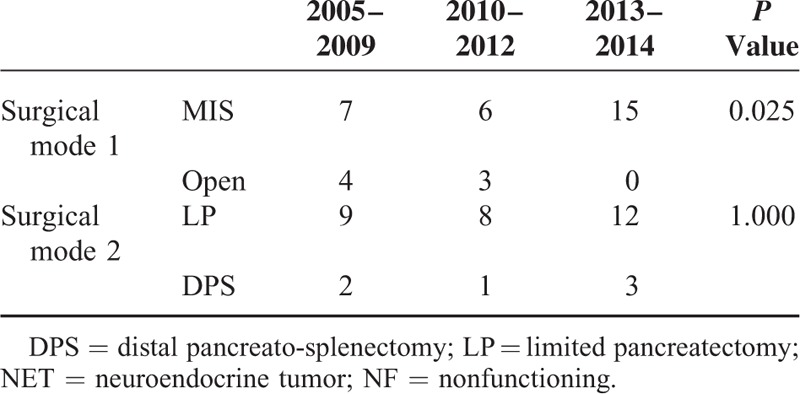
Chronological Change of Surgical Mode to Treat G1 NF-NET of the Left-Sided Pancreas

### Oncologic Outcomes Following Pancreatectomy for NF NET-G1 of the Left Side of the Pancreas

**FIGURE 4:**
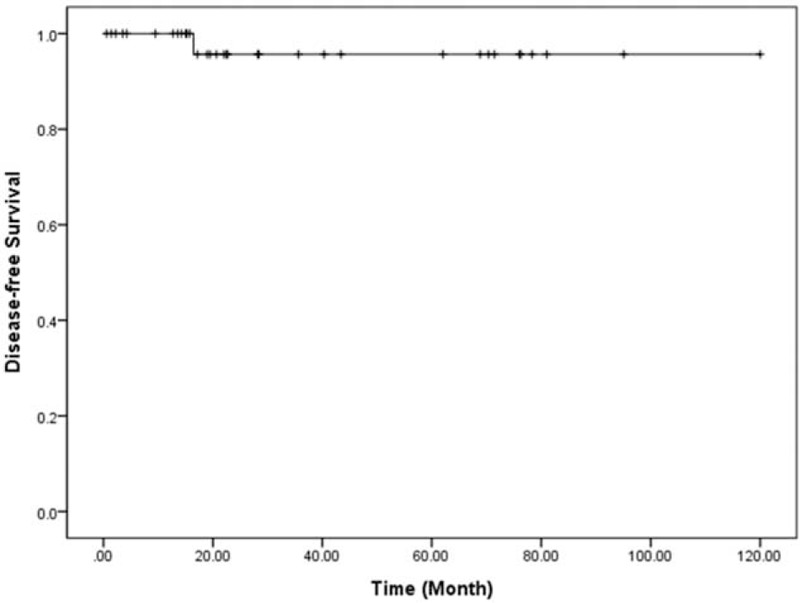
Disease-free survival of resected NF NET-G1 of the left side of the pancreas. NET = neuroendocrine tumor; NF = nonfunctioning.

**FIGURE 5:**
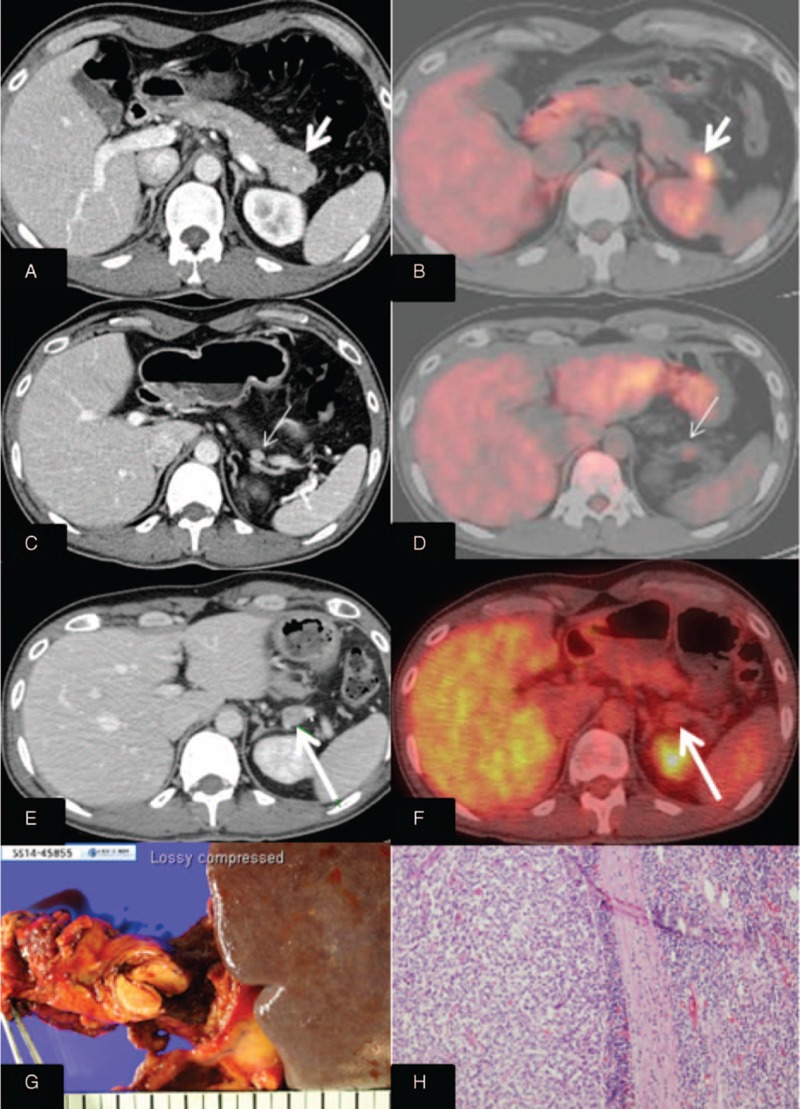
One case of recurrence following robotic spleen-preserving distal pancreatectomy.

All patients were found to have margin-negative resection. During the follow-up period, a mean of 37.5 months (range, 0.6–120.0 months), disease-free survival was 36.5 months on average (95% confidence interval [CI]: 25.9–47.1) (Figure 4). Only 1 patient experienced local recurrence (Figure 5). Upon retrospective reevaluation of this recurrence, we noted suspicious regional lymph node metastasis before surgery (cN1) that was underestimated in the preoperative diagnostic stage. The patient subsequently underwent laparoscopic distal pancreatosplenectomy. Pathologic examination of the lesion revealed NET-G1 lymph node metastasis. Excluding this patient, no systemic or local recurrence was noted. There was no disease-specific mortality during the follow-up period either.

### Oncologic Impact of Pathologic Characteristics and Limited Pancreatectomy in Resected NF NET-G1s of the Left Side of the Pancreas

**FIGURE 6:**
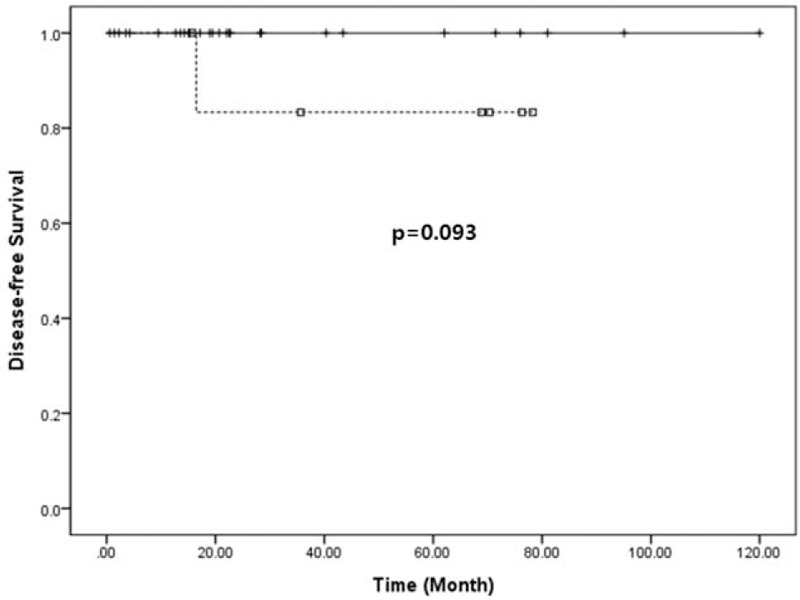
Survival differences according to lymphovascular invasion.

**FIGURE 7:**
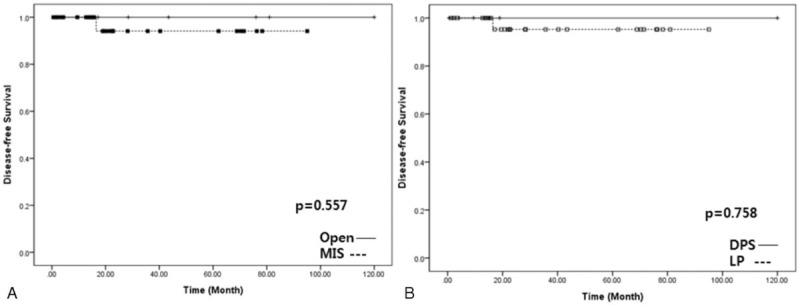
Oncologic impact of minimally invasive and limited pancreatectomy.

Radiologic tumor size (>2.5 cm) was closely associated with lymphovascular invasion (*P* = 0.014, Table 4). Disease-free survival was marginally significant according to lymphovascular invasion (*P* = 0.093, Figure 6). There was no significant survival differences between minimally invasive pancreatectomy and open pancreatectomy (*P* = 0.557, Figure 7A). In addition, no adverse oncologic impact of limited pancreatectomy was noted in treating NF NET-G1 of the left side of the pancreas (*P* = 0.758, Figure 7B).

**TABLE 4:**
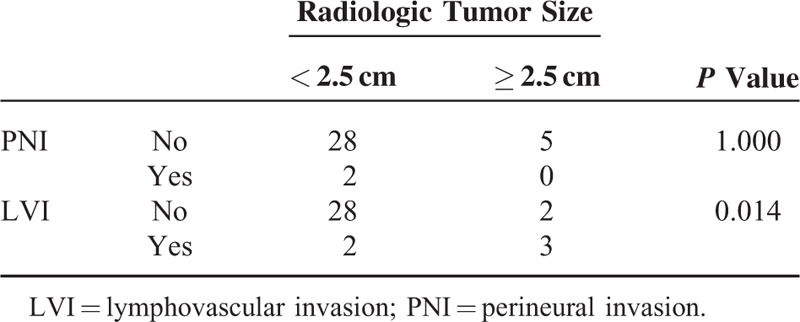
Correlation Between Pathologic Characteristics and Radiologic Tumor Size

## DISCUSSION

Several studies suggest that regional lymph node metastasis is related to poor oncologic outcomes in treating NF NETs. Reporting on surgical outcomes for pancreatic NETs, Fisher et al^7^ found grade 3, lymph node metastasis, and distant metastasis to be independent prognostic factors in patients with neuroendocrine neoplasms. Of note, they found that even small NET-G1s (median, 1.5 cm with range, 0.4–11 cm) accompanied lymph node metastasis in 16.7% of the patients (28 out of 168 patients). Hashim et al^15^ observed that approximately 40% of their patients (50 out of 136 patients) had lymph node metastasis, which was associated with larger tumors, tumors of the pancreatic head, lymphovascular invasion, high Ki-67 (>20%), and poor disease-free survival. They concluded that regional lymphadenectomy should be included in patients undergoing pancreatic resection for pancreatic NETs. Tsutsumi et al^16^ showed that NETs of more than 1.5 cm in size are associated with a high incidence of lymph node metastasis (25%, 10 out of 40 patients), suggesting that lymph node dissection should be recommended in NETs larger than 1.5 cm. In resectable PNETs, Han et al^17^ also found that CK-19 and KIT expression could predict clinical behavior, advanced stage, and regional lymph node metastasis.

Although important data support the adverse oncologic impact of lymph node metastasis and rationale for lymph node dissection, the proportion of NF NETs in these studies is limited,^16^ and no subgroup analysis has focused on NF NET-G1.^7,15,16^ In addition, none have attempted to delineate recurrence patterns.^7,15^ In order to suggest the importance of lymph node dissection in NF-NET of the pancreas, it should be clearly shown that loco-regional recurrence is much higher in cases of omitting lymph node dissection. However, the literature does not support this expectation.^18^ Recently, Tsutsumi et al^19^ evaluated risk factors for recurrence after curative resection of well-differentiated pancreatic NET based on the new grading classification. While their univariate analysis demonstrated lymph node metastasis as a significant risk factor for tumor recurrence (*P* = 0.0004), multivariate analysis did not (hazard ratio = 0.32 [95% CI: 0.01–7.98], *P* = 0.4948). Brinbaum et al^20^ also investigated clinicopathological factors influencing survival of NET of the pancreas and showed that lymph node metastasis is not associated with poor disease-free survival (hazard ratio = 1.11 [95% CI: 0.57–2.16], *P* = 0.74). Instead, they concluded synchronous liver metastasis (hazard ratio = 3.11 [95% CI: 1.15–8.39], *P* = 0.025) and portal vein resection (hazard ratio = 15.8 [95% CI: 2.60–96.40], *P* = 0.002) were found to be independent prognostic factors for predicting tumor recurrence. Moreover, Gratian et al^21^ attempted to determine whether extent of surgery or lymph node dissection is associated with overall survival in 1854 patients with NF NETs <2 cm. Surprisingly, multivariate analysis demonstrated that type of surgery and lymphadenectomy were not associated with overall survival. Bilimoria et al^22^ also performed an analysis of a large cohort of patients from the National Cancer Database (N = 3851). They observed lymph node metastasis in 1384 patients (52.8%); however, lymph node status did not impact survival in multivariate analysis (hazard ratio = 1.18 [95% CI: 0.96–1.45], *P* = 0.12). These all suggest that the oncologic impact of regional lymph node metastasis is still debatable in NETs of the pancreas and put into question the rationale of routine regional lymph node dissection for treating NF-NET of the pancreas.

According to our results, excluding only 1 patient whose preoperative lymph node metastasis was underestimated on preoperative computed tomography scan, no patients with function-preserving limited pancreatectomy and minimally invasive pancreatectomy experienced tumor recurrence during the follow-up period (mean 37.5 months; range of 0.6–120.0 months). Partelli et al^23^ investigated clinical predictors of lymph node involvement in NF NET of the pancreas. They concluded that NF NET-G1 has a very low risk of lymph node metastasis in the absence of radiological signs of node involvement. Therefore, careful preoperative radiologic evaluation for regional lymph node metastasis should be important in choosing surgical option for treating NF NET-G1 of the pancreas.

Our data further discredit the oncologic role of lymph node retrieval in NF NET-G1s of the left side of the pancreas. No survival difference between distal pancreatosplenectomy and limited pancreatectomy (Nx-pancreatectomy) posits the following points: the incidence of lymph node metastasis in NF NET-G1s of the left side of the pancreas may be very low, and the oncologic impact of lymph node metastasis may be overestimated in NF NET-G1. In our data, Nx-pancreatectomy (limited pancreatectomy) was undertaken in 29 patients (82.9%). During long-term follow-up, tumor recurrence was found in only 1 patient in whom lymph node metastasis was preoperatively underestimated. Interestingly, even the patient with regional lymph node metastasis (recurred case, Figure 5) showed a Ki-67 index of <1%, raising doubts about the oncologic impact of lymph node metastasis. In addition, presuming that lymph node dissection in NF NET-G1s plays a therapeutic role, it would be expected that oncologic outcomes for patients undergoing regional lymphadenectomy with lymph node metastasis would be similar to those for patients undergoing the same treatment without lymph node metastasis. However, as the literature suggests,^21,22^ poor oncologic outcomes even after regional lymph node dissection suggest that lymph node metastasis is associated more with aggressive tumor biology than extent of treatment. Accordingly, the oncologic impact of lymph node metastasis may not be a correctable by regional lymph node dissection.

As an important practical issue, it is impossible to know exact histologic grades without pathological examination in treating NF NETs of the pancreas. However, thanks to advanced radiologic techniques, we may be able to predict not only the status of lymph node metastasis but also tumor grade in preoperative settings. Kim et al^24^ reported that NET-G1 could be differentiated from NET-G2 or pancreatic NEC based on preoperative magnetic resonance imaging images. They demonstrated that NET-G2 and NEC exhibit specific findings, such as ill-defined borders (*P* = 0.001) and hypo-signal intensity on venous- and delayed-phase (*P* = 0.016), with high predictive value for discriminating NET-G1 from G2 (*P* = 0.007).

It is well known that most NF NET-G1s <2 cm are not eligible for surgery if they are asymptomatic and incidental. However, according to the 2010 WHO classification of tumors, even the smallest of tumors are given a cancer code.^9^ Clinical guidelines for gastroenteropancreatic NETs in Japan indicate surgical resection should be performed even in tumors <2 cm in size.^25^ Bettini et al^26^ also reported that NF-NETs larger than 2 cm increased the chances of malignancy, and it was also suggested that tumors smaller than 1 cm in size could be malignant.^27^ Gratian et al^21^ further showed that 11% of tumors smaller than 0.5 cm exhibit distant metastasis. Accordingly, size is not an accurate predictor of malignancy. Nevertheless, although several studies have failed to reveal any association between tumor size and prognosis,^28,29^ tumor size is thought to be an important clinical parameter in choosing between surgical modalities in treating NF NET of the pancreas. We previously reported that malignant behavior of NF NET of the pancreas is associated with tumor size ≥3 cm.^30^ Our current observation showed that larger tumors (>2.5 cm) are marginally associated with tumor recurrence (*P* = 0.075) and lymphovascular invasion in NF NET-G1 of the left side of the pancreas (*P* = 0.014). Additionally, radiologic tumor size, with a cut-off value of 2.5 cm, predicted future tumor recurrence with 100% sensitivity and a false positive rate of 11.8% (data not shown), requiring future validation study in larger number of patients. All together, these suggest that routine lymph node dissection is not recommended and that lymph node dissection should be selected upon preoperative imaging. Tailor-made surgical approaches also need to be considered.

Based on our experiences and the following rationale, we concluded that the oncologic impact of lymph node metastasis in NF NET-G1 of the left side of the pancreas may be overestimated: Most NET-G1s are relatively small. Less frequent lymph node metastasis is noted in NF NET of the left side of the pancreas, especially in the absence of radiologic signs of node involvement.^23^ The oncologic impact of lymph node metastasis is still controversial.^22,31^ Except for 1 patient with underestimated lymph node metastasis, no regional recurrence was noted even after limited pancreatectomy (current observation). Recent studies suggest that incidental pancreatic NETs are relatively small and nonaggressive^32,33^: small asymptomatic NETs usually exhibit minimal or no growth over many years; therefore, a nonoperative follow-up policy may be useful in some cases.^34^ Minimally invasive enucleation, central pancreatectomy, and left-sided pancreatectomy are feasible and safe.^35–37^ Notwithstanding, large volume-based studies are needed to validate the current suggestion and clinical observations. In addition, considering the low proliferative power of NF NET-G1 (Ki67 index <1%), more long-term follow-up study should conducted to reach a concrete conclusion.

In conclusion, our results further put into doubt the oncologic impact of lymph node metastasis in NF NET-G1 of the left side of the pancreas. Therefore, routine conventional distal pancreatectomy with splenectomy for retrieving regional lymph nodes may be too extensive in NF NET-G1. Minimally invasive and function-preserving pancreatectomy is recommended in well-selected NF NET-G1 as long as there are relevant predicting models to predict tumor grade before surgery. Future studies to help realize tailored surgical approaches for use in treating NF NET of the left side of the pancreas are warranted.
